# Glomerular Endothelial Cell Injury and Focal Segmental Glomerulosclerosis Lesion in Idiopathic Membranous Nephropathy

**DOI:** 10.1371/journal.pone.0116700

**Published:** 2015-04-15

**Authors:** Megumi Morita, Akiko Mii, Akira Shimizu, Fumihiko Yasuda, Jun Shoji, Yukinari Masuda, Ryuji Ohashi, Kiyotaka Nagahama, Tomohiro Kaneko, Shuichi Tsuruoka

**Affiliations:** 1 Department of Nephrology, Nippon Medical School, Tokyo, Japan; 2 Department of Analytic Human Pathology, Nippon Medical School, Tokyo, Japan; 3 Department of Nephrology, George Washington University Medical Center, Washington, District of Columbia, United States of America; 4 Division of Diagnostic Pathology, Nippon Medical School Hospital, Tokyo, Japan; Fondazione IRCCS Ospedale Maggiore Policlinico & Fondazione D’Amico per la Ricerca sulle Malattie Renali, ITALY

## Abstract

**Background:**

Focal segmental glomerulosclerosis (FSGS) lesions have often been discussed as a negative predictor in idopathic membranous nephropathy (MN). The mechanism of the development of FSGS lesion in MN is still uncertain.

**Methods:**

From 250 cases of MN, 26 cases contained FSGS lesion. We compared the clinicopathological characteristics between MN cases with FSGS lesion [MN-FSGS(+)] and MN without FSGS lesion [MN-FSGS(−)], matched for gender, age, stage of MN.

**Results:**

The glomerular filtration rate (eGFR) was significantly lower in MN-FSGS(+) cases compared to MN-FSGS(−), although nephrotic syndrome, hematuria, and systolic blood pressure levels were not significantly different between the two groups. Pathologically, glomeruli in MN-FSGS(+) cases showed narrowing and loss of glomerular capillaries with separating from GBM or disappearance of CD34+ endothelial cells, and accumulation of extracellular matrix (ECM) in capillary walls, indicating the development of glomerular capillary injury. These findings of endothelial injury were seen even in MN-FSGS(−) cases, but they were more prominent in MN-FSGS(+) than MN-FSGS(−) by computer assessed morphometric analysis. In MN-FSGS(+) cases, 44 out of 534 glomeruli (8.2%) contained FSGS lesions (n = 31, NOS lesion; n = 13, perihilar lesion). Significant thickness of GBM with ECM accumulation was evident in MN-FSGS(+) cases. Podocyte injury with effacement of foot processes was also noted, but the expression of VEGF on podocytes was not different between the two groups, which suggests that the significant thickness of capillary walls may influence the function of VEGF from podocyte resulting in the glomerular capillary injury that contribute to the development of FSGS lesion in MN.

**Conclusion:**

Glomerular capillary injury was seen in all MN cases. Furthermore, the prominent injuries of glomerular capillaries may be associated with the deterioration of eGFR and the formation of FSGS lesions in MN.

## Introduction

Idiopathic membranous nephropathy (MN) is one of the most common causes of nephrotic syndrome in adults [1,2]. The course of MN is quite variable, with an estimated one third of patients undergoing spontaneous remission of proteinuria, another third with persistent proteinuria, and the remaining third progressing to end-stage renal failure [2,3]. Because of such variable natural history of MN, the identification of parameters that predict the prognosis of MN is important in order to select appropriate treatment, conservative or immunosuppressive therapy.

Several clinical and pathological parameters, including focal segmental glomerulosclerosis (FSGS), were reported as poor prognostic indicators of MN [4–8]. However, it is still open to debate if the coexistence of FSGS lesion can predict the prognosis of MN [8–11]. At least clinicopathological characteristics of MN cases with FSGS lesion [MN-FSGS(+)] are still uncertain, although several studies have shown a trend toward lower renal function in MN-FSGS(+) patients, hypertension, and high serum creatinine at the time of biopsy [12–14].

Furthermore, the etiology and pathogenesis of FSGS lesion in MN has not been clarified. FSGS lesion in primary and secondary FSGS is considered to be mediated by podocyte injury, termed podocytopathy [15,16]. On the other hand, morphological FSGS lesion in preeclampsia and malignant hypertension is probably mediated by the combination of glomerular endothelial cell injury and podocyte injury [17,18].

In the present study, in order to clarify the clinicopathological characteristics of MN-FSGS(+) cases, and the mechanism of the development of FSGS lesion in MN, we examined retrospectively the cases of MN with and without FSGS, focusing on the clinical characteristics, glomerular endothelial and capillary injury, thickening of glomerular capillary walls with the accumulation of extracellular matrix (ECM), and the expression of VEGF in podocytes.

## Materials and Methods

### Ethics statement

The study was carried out in accordance with the Declaration of Helsinki and approved by the institutional review board of Nippon medical school. Written consent for using the samples for research purposes was obtained from all patients.

### Case selection

We selected idiopathic MN cases (n = 250) from a series of biopsies in our department from 1994 to 2012. Secondary causes of MN such as malignancy, lupus erythematosus, hepatitis B and C, rheumatoid arthritis, medications, and toxic agents were excluded. From 250 cases of idiopathic MN, we selected 26 cases whose biopsies contained FSGS lesion. We also selected 26 cases of MN without FSGS lesion [MN-FSGS(−)], matched for gender, age, stage of MN, similar to previous study by Wakai and Magil [10]. We compared the clinicopathological characteristics between MN cases with and without FSGS lesion.

### Clinical Findings, Laboratory Data, and Pathology

Age, gender, nephrotic syndrome, systolic blood pressure, microscopic hematuria, and estimated glomerular filtration rate (eGFR) at the time of biopsy of 52 patients were examined retrospectively using clinical records.

Kidney biopsies were evaluated by light microscopy, immunohistochemistry, and electron microscopy. Sections for light microscopy were prepared from formalin-fixed paraffin-embedded tissue and stained with hematoxilin and eosin (H&E), periodic acid-Schiff (PAS), Masson trichrome (Masson), and periodic acid silver methenamine (PAM). The biopsies were evaluated in detail for the following features: total number of glomeruli, global sclerosis, glomeruli exhibiting FSGS lesion, characterization of FSGS lesion using the criteria of Columbia classification of FSGS [19], the extent of interstitial fibrosis, and the degree of arteriosclerosis. Interstitial fibrosis was graded semiquantitatively from 0 to 3 (1: area of interstitial fibrosis of 5∼24%, 2: fibrosis of 25∼49%, 3: fibrosis of 50% or greater). Degree of arteriosclerosis was assigned scores of 0, 1, 2, or 3 for no change, mild, moderate, or marked intimal sclerosis and/or hyalinosis, respectively.

Immunofluorescence studies were performed to make the diagnosis of idiopathic MN. Immunohistochemistry was used to detect cells by specific markers including CD34 (endothelial cells: NU-4A1, Nichirei Bioscience, Tokyo, Japan), α-smooth muscle actin (αSMA; activated mesangial cells: 1A4, Dako, Glostrup, Denmark), CD 68 (macrophages: PG-M1, Dako), CD3 (T cells: A0452: Dako) and MPO (neutrophils: A398, Dako). To evaluate the expression of VEGF (A-20, Santa Cruz Biotechnology, Dallas, Texas) on podocytes, staining intensity of VEGF in glomeruli was scored semiquantitatively from 0 to 3 (0: no visible staining; 1: faint staining; 2: moderate intensity with multifocal staining; 3: intense diffuse staining).

The electron microscopic studies were performed in all cases of MN with or without FSGS lesion. Ultrathin sections from Epon-embedded tissue samples after fixation in 2.5% glutaraldehyde and postfixation in 1% osmium tetroxide were stained with uranyl acetate and lead citrate and examined with Hitachi H7500 electron microscope (Hitachi, Ibaraki, Japan). MN was staged from 1 to 4 with electron microscopy according to the Ehrenreich and Churg’ s ultrastructural criteria [11,20].

The thickness of glomerular capillary walls was measured using the electron microscopy images as the distance between podocytes and glomerular endothelial cells, which include subepithelial deposits, glomerular basement membrane (GBM), and subendothelial space. The thickness of glomerular capillary walls was measured at 10 randomly selected points of all glomerular capillaries, and the mean ± SE was calculated.

### Computer-assisted Morphometric Analysis

**Fig 1:**
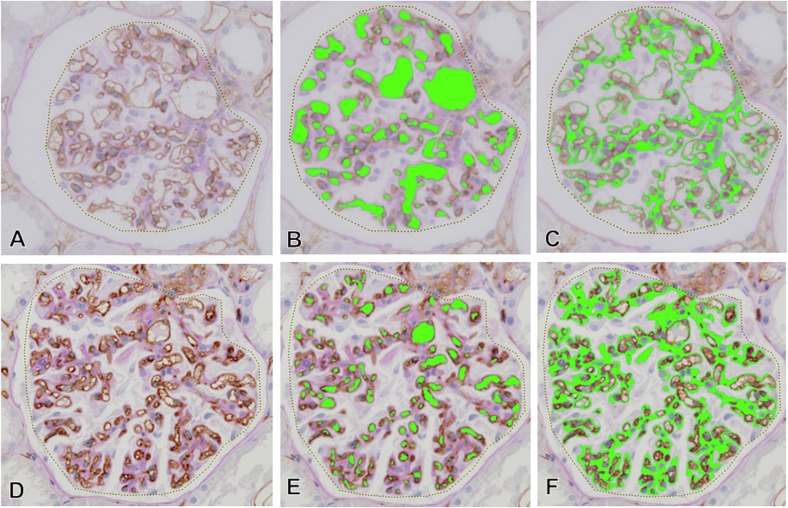
The areas of glomerular capillaries and glomerular ECM in computer-assessed morphometric analysis. The area of glomerular tuft (dotted line in A and D), the area and number of glomerular capillaries (green areas in B and E), and the area of glomerular ECM (green areas in C and F) in each glomerulus were assessed by computer-assisted image analyzer. In the nearly normal glomeruli in light microscopic findings (A-C), large glomerular capillary area was noted with minimal ECM accumulation. In contrast, the area of glomerular capillaries decreased with narrowing glomerular capillaries and the accumulation of glomerular ECM in glomerulus in the development of glomerular sclerosis (D-F).

We assessed the areas of glomerular capillaries and glomerular ECM including mesangial matrix and thickening of capillary walls in each glomerulus in the sections with CD34 immunostaining (glomerular capillaries) and PAS counterstain (ECM) by a computer-assisted image analyzer (Win Roof, Mitani Corp., Japan). The area of glomerular capillaries was detected as the space enclosed by CD34-positive endothelial cells in glomeruli, and the area of glomerular ECM was detected as PAS counterstain-positive area in glomeruli (Fig. 1). We measured the area of glomerular tuft, the area of glomerular capillaries, and the area of ECM in each glomerulus. In addition, we also counted the number of glomerular capillary lumens in each glomerulus. 96 glomeruli, 86 glomeruli, and 102 glomeruli that contained a vascular pole and large glomerular area were selected from 26 MN-FSGS(+) cases, 26 MN-FSGS(−) cases, and age-matched 21 cases with minor glomerular abnormalities as control, respectively.

### Statistical Analysis

Statistical analysis was performed using by StatMate IV for Windows Ver.4.01. The Student’s T test, the chi-square test, and the Mann-Whitney U test were utilized where appropriate. Correlations were calculated using Spearman’s correlation test. P<0.05 was considered statistically significant for all tests.

## Results

### Clinical Characteristics

In 250 MN cases, 26 cases (16 Male, 10 Female) (10.4%) were accompanied by FSGS lesion. The clinical characteristics of MN-FSGS(+) and MN-FSGS(−) cases summarized in Table 1. The average age of MN-FSGS(+) cases was 62.4 ± 9.8 years old. eGFR in MN-FSGS(+) (57.4 ± 18.1 ml/min/1.73m^2^) was significantly lower compared to MN-FSGS(−) (68.4 ± 17.9 ml/min/1.73m^2^, P<0.05). Despite lack of significant difference, higher percentage of nephrotic syndrome developed in MN-FSGS(+) (77%) compared with MN-FSGS(−) (54%, p = 0.08). In regards to the age, gender, systolic blood pressure, and microscopic hematuria, there were no significant differences between the two groups.

**Table 1:** Clinical characteristics at the time of biopsy in MN cases with or without FSGS lesion.

	MN with FSGS	MN without FSGS
	lesion (n = 26)	lesion (n = 26)
Age*	62.4 ± 9.8	59.0±12.7
Gender***	male; n = 16	male; n = 17
	female; n = 10	female; n = 9
Nephrotic	77% (n = 20)	54% (n = 14)
syndrome***				
Systolic blood	137.1 ± 15.7	134.0 ± 18.7
pressure (mmHg)**		
Urinary occult blood	50% (n = 13)	35% (n = 9)
(≧2+)***		
eGFR	57.4 ± 18.1	68.4 ± 17.0
(ml/min/1.73m2)*		

* Student’s t-test

** Mann-Whitney U test

*** Chi-square test

abbreviations: MN, idiopathic membranous nephropathy; FSGS, focal segmental glomerulosclerosis

eGFR, estimation of glomerular filtration rate

### The Morphological Characteristics of MN with/without FSGS Lesion

**Fig 2:**
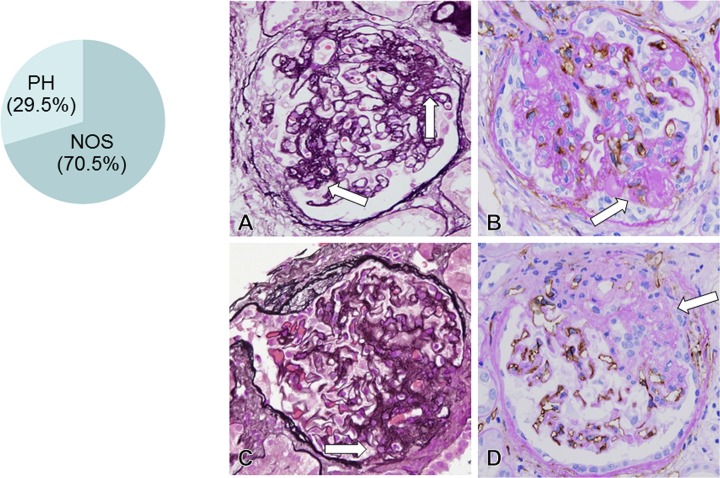
FSGS lesion in idiopathic membranous nephropathy(MN) (A, C: PAM stain, x600; B, D: CD34 stain, x600). The biopsy samples from 26 MN-FSGS(+) cases included 534 glomeruli except for 55 global sclerotic glomeruli. In 534 glomeruli, 44 glomeruli (8.2%) had FSGS lesion, consisted of not otherwise specified (NOS) lesion type (31 glomeruli, 70.5%) and perihilar (PH) lesion type (13 glomeruli, 29.5%). In glomeruli with NOS type of FSGS lesion (arrow in A), FSGS lesion was noted in areas other than perihilar region, with hyalinosis and adherence lesion between tuft and Bowman’s capsule. In FSGS lesion (arrow in B), loss of CD34+ capillaries was evident with mesangial ECM accumulation. In glomeruli with PH type of FSGS lesion (arrow in C), perihilar sclerosis with hyalinosis was noted with loss of CD34+ glomerular capillaries (arrow in D) and mesangial ECM accumulation.

In 26 MN-FSGS(+) cases, there were 534 glomeruli except for 55 global glomerular sclerosis, and 44 glomeruli (8.2%) contained FSGS lesion in glomeruli. According to the criteria of FSGS lesion in Columbia classification of FSGS [19], FSGS lesion in the present study was classified into NOS lesion (70%: n = 31) and perihilar (PH) lesion (30%: n = 13) (Fig. 2). Collapsing, cellular, and TIP variant lesions of FSGS were not detected in the present study.

**Fig 3:**
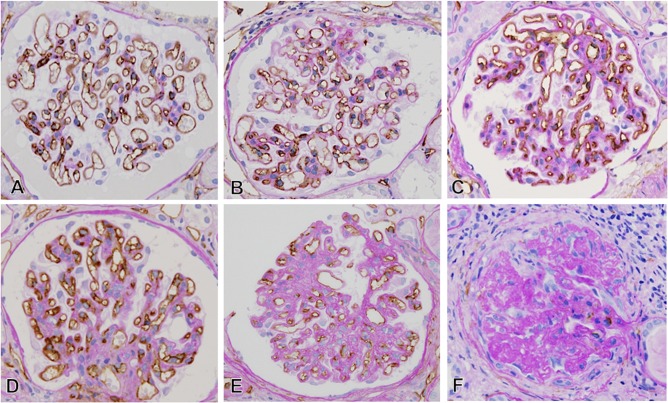
Several glomerular capillary alterations in cases of idiopathic membranous nephropathy without FSGS lesion (CD34 stain, x600). In the glomeruli with minimal glomerular abnormalities by light microscopy (A), nearly normal glomerular capillary network was identified without ECM accumulation. During the development of capillary narrowing in small glomerular area (B), segmental glomerular area (C), and global glomerular area (D and E), ECM accumulated in mesangium and capillary walls in glomeruli. In global sclerotic glomeruli (F), marked and complete loss of glomerular capillaries was noted with massive accumulation of ECM in glomeruli.

**Fig 4:**
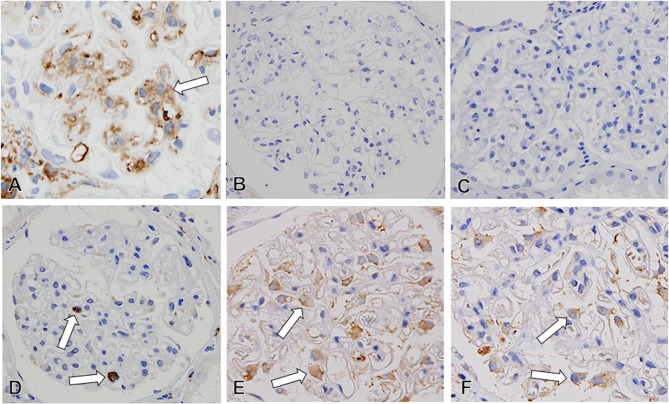
The activation of mesangial cells, infiltration of inflammatory cells in glomeruli, and the expression of vascular endothelial growth factor (VEGF) on podocytes in idiopathic membranous nephropathy (MN) (A: αSMA, B: MPO, C: CD3, D: CD68, E-F: VEGF, x600). In MN, the αSMA-positive activation of mesangial cells (arrow in A) was noted with mesangial ECM accumulation. In glomeruli, although infiltration of MPO-positive neutrophils (B) and CD3-positive T cells (C) was hardly observable, CD68-positive macrophages (arrow in D) frequently infiltrated the glomeruli. Similar degree of expression of VEGF (arrow) was evident on podocytes in both MN cases with (E) and without (F) FSGS lesion.

The endothelial cells in glomerular capillaries were assessed by immunostaining with CD34, which was expressed on all of endothelial cells. In MN-FSGS(+) cases, loss of glomerular capillaries with absence of the CD34-positive glomerular endothelial cells was evident in FSGS lesion, in both NOS and PH lesions (Fig. 2). In addition, narrowing and reduced number of glomerular capillaries with ECM accumulation were noted in the areas of glomeruli where FSGS lesion were excluded and in glomeruli that don’t contain FSGS lesion. Even in MN-FSGS(−) cases, similar glomerular capillary injury was seen in glomeruli (Fig. 3). In morphologically normal glomeruli, almost normal CD34-positive capillary network was noted in glomeruli. However, the narrowing and decreasing number of CD34-positive glomerular capillaries occur with accompanying ECM accumulation, and the global sclerosis of glomeruli eventually developed with complete loss of glomerular capillaries and massive ECM accumulation. In the damaged glomeruli, narrowing of glomerular capillaries was sometimes accompanied by several degrees of mesangial expansion and mesangial hypercellularity. In the expanded mesanigal lesions, αSMA-positive activation of mesangial cells was evident in mesangial interposition and mesangial hypercellularity lesions (Fig. 4).

### Ultrastructure of Capillaries in MN

**Fig 5:**
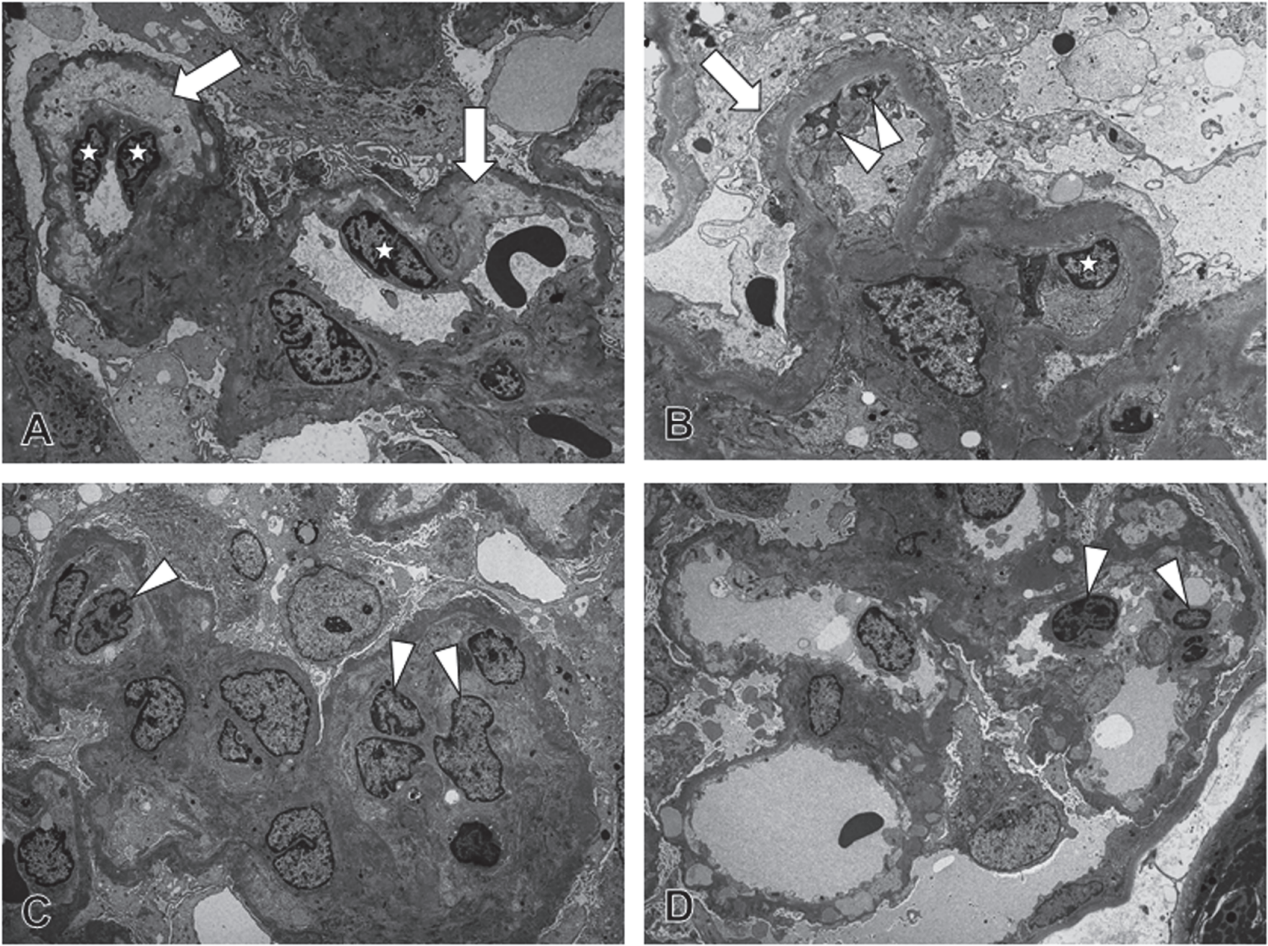
The injury of the glomerular endothelial cells and podocytes in idiopathic membranous nephropathy (MN). (A) In MN-FSGS(+) cases, an increase in the number of endothelial cells, swelling of the nuclei and cytoplasm, loss of fenestra, and widening of the subendothelial space were noted, suggesting the development of glomerular endothelial cell injury. (B) Narrowing of the capillary lumen was seen with foot process effacement of podocytes, widening of subendothelial space, and mesangial interposition, suggesting the presence of podocyte injury and the findings of reaction of glomerular endothelial cell injury. (C) Infiltration of monocytes and macrophages was noted in glomerular capillary lumens and subendothelial spaces with swelling of the nuclei and cytoplasm and loss of fenestra in glomerular endothelial cells, suggesting the glomerular endothelial cell injury and macrophage infiltration. (D) In MN-FSGS(−) cases, segmental glomerular endothelial cell damage was detected with infiltration of monocyte and macrophages, thickening of glomerular capillary walls, and loss of foot processes of podocytes, suggesting that, although the severity was mild, injuries of glomerular endothelial cells and podocytes were developed in glomeruli even in MN-FSGS(−) cases.

In MN-FSGS(+), narrowing of glomerular capillary lumens were noted with damaged endothelial cells, characterized by the increase in the number of endothelial cells, swelling of the nuclei and cytoplasm, and loss of fenestra of endothelial cells (Fig. 5). In addition, narrowing of the capillary lumens was also noted with widening of the subendothelial space, ECM accumulation, mesangial interposition, and monocyte and macrophage infiltration. The effacement of foot processes was also seen in podocytes. These ultrastructual findings demonstrated that damage of glomerular endothelial cells and podocytes developed in glomeruli in MN-FSGS(+). In MN-FSGS(−) cases, minor segmental glomerular endothelial cell damage was detected with infiltration of macrophages and segmental loss of foot processes of podocytes.

### Computer-assisted Morphometric Analysis

We assessed the degree of narrowing of glomerular capillaries that is most likely associated with glomerular endothelial injury and the accumulation of ECM in each glomerulus with a computer-assisted morphometric analysis (Fig. 1).

**Fig 6:**
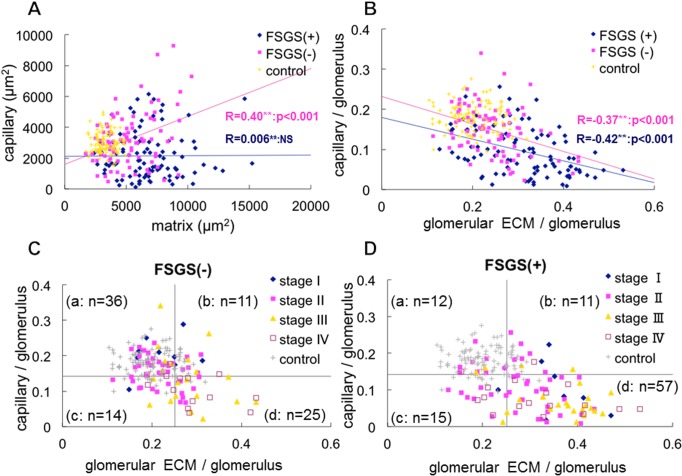
The relationship between the areas of glomerular capillaries and glomerular ECM in glomerular cross section in idiopathic membranous nephropathy (MN) by computer-assessed morphometric analysis. (A) The areas (μm^2^) of glomerular capillaries (X-axis) and ECM (Y-axis) were measured in MN-FSGS(+) cases (blue rhombus), MN-FSGS(−) cases (red square), and minor glomerular abnormalities in control (yellow x). In MN cases with or without FSGS lesion, increase of glomerular capillary area and/or glomerular ECM area was evident, suggesting the development of glomerular hypertrophy. In MN-FSGS(−) cases, the area of capillaries was positively correlated with the area of ECM in glomeruli. In contrast, there was no correlation between the areas of capillaries and ECM in glomeruli in MN-FSGS(+), suggesting the development of decrease of glomerular capillary area with increase of ECM area in enlarged glomeruli. (B) To avoid the effects of the variation in the size of glomeruli, the areas of glomerular capillaries and ECM in glomeruli were adjusted by using the area of glomerular capillaries / the area of glomerular tuft, and the area of glomerular ECM / the area of glomerular tuft. Regardless of the presence or absence of FSGS lesion, the areas of glomerular capillaries and ECM were negatively correlated, indicating that the narrowing and decrease of glomerular capillaries was associated with the accumulation of ECM. (C, D) We examined the areas of glomerular capillaries and ECM in glomeruli in each stage of MN (stage I to IV) in cases without FSGS lesion (C, FSGS −) and with FSGS lesion (D, FSGS +). The horizontal boundary is set at the average of glomerular capillary area of all glomeruli in control and MN cases with and without FSGS lesion. The longitudinal boundary is set at the average of glomerular ECM area of all glomeruli in control and MN cases with and without FSGS lesion. In accordance with the setting the boundary for the average of the areas of glomerular capillaries and glomerular ECM, all glomeruli were divided into 4 categories; a: large capillary area with minimal accumulation of ECM, b: large capillary area with marked accumulation of ECM, c: decrease of glomerular capillary area with minimal accumulation of ECM, and d: decrease of glomerular capillary area with marked accumulation of ECM. (n = number) is the number of cases of MN with and without FSGS lesion in each category. In MN-FSGS(−) cases (C), many cases, especially stage I and stage II, were present in category a (36/86: 41.9%). In contrast, the most common category in MN-FSGS(+) cases (D), was d (57/95: 60.0%), and many cases of stage III (18/24: 75.0%) and IV (11/17:64.7%) fell into this category. These findings indicate that the decrease of glomerular capillaries with increase of glomerular ECM area was more prominent in MN-FSGS(+) than those in MN-FSGS(−) cases.

**Fig 7:**
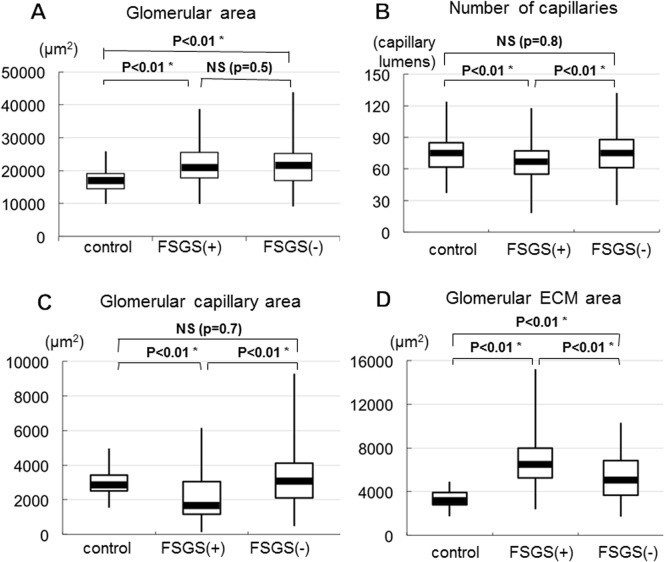
Box and Whisker plot of the glomerular tuft area (enlarged glomeruli) (A), the number of glomerular capillaries (B), the glomerular capillary area (C), and the glomerular ECM area (D) in cases of minor glomerular abnormalities (control), MN cases with FSGS lesion (FSGS +) and without FSGS lesion (FSGS −). (A) The enlargement of glomerular tuft area (glomerular hypertrophy) was evident in both MN cases with and without FSGS lesion compared to control. However, there was no significantly difference in glomerular tuft area in MN cases with and without FSGS lesion. (B) The decreased number of glomerular capillaries was noted in MN-FSGS(+) compared to control and MN-FSGS(−), suggesting the prominent loss and reduced glomerular capillaries in MN-FSGS(+). (C) The reduced area of glomerular capillaries was noted in MN-FSGS(+) cases compared to control and MN-FSGS(−), indicating the narrowing of glomerular capillaries in MN-FSGS(+). There was no significant difference in glomerular capillary area in control and MN-FSGS(−) cases. (D) The increase of glomerular ECM area was observed in MN with or without FSGS lesion compared to control, suggesting the development of enlarged glomeruli and the narrowing glomerular capillaries with ECM accumulation in MN cases.

In MN cases with or without FSGS lesion, the area of glomerular ECM and/or glomerular capillaries increased (Fig. 6A), indicating the development of enlarged glomeruli. In fact, glomerular tuft area was larger in MN cases than in control with minor glomerular abnormalities (Fig. 7A), although there was no significant difference of enlarged glomeruli between MN cases with and without FSGS lesion. In addition, in MN-FSGS(−) cases, the area of the capillaries was positively correlated with the area of ECM in glomeruli (Fig. 6A), indicating the enlargement of glomeruli with both increased areas of glomerular capillaries and glomerular ECM. In MN-FSGS(+), there was no correlation between the areas of glomerular capillaries and glomerular ECM, which may indicate the narrowing and obstruction of glomerular capillaries with accumulation of ECM in enlarged glomeruli. The number and the area of glomerular capillaries were significantly less in MN-FSGS(+) cases than in MN-FSGS(−) (Fig. 7B, C).

We examined the propotion of glomerular capillaries and ECM in glomeruli to glomerular tuft area to avoide the effects of enlarged glomeruli. In MN cases with or without FSGS lesion, the area of glomerular capillaries was negatively correlated with the area of ECM (Fig. 6B), indicating that the decrease in glomerular capillary area was associated with increase in glomerular ECM area in all MN cases, despite the presence or absence of FSGS lesion. However, the decrease of glomerular capillary area with increase of glomerular ECM area was more significantly prominent in MN-FSGS(+) than MN-FSGS(−) cases (Figs. 6B-D, 7C, D).

### The Mechanisms of Glomerular Endothelial Cell Injury in MN

We examined the mechanism of glomerular endothelial cell injury in MN. First, we examined the inflammatory cell infiltration in glomeruli. Infiltration of neutrophils and T lymphocytes in glomeruli were hardly detectable, but several macrophages infiltrated the glomeruli. From this observation, we concluded that glomerular endothelial cell damage was not mediated by infiltration of neutrophils and T cells, but due to macrophage infiltration that may be associated with the reaction to the endothelial cell injury in glomeruli.

We examined the expression of vascular endothelial growth factor (VEGF), which is produced mainly by glomerular podocytes, and has a crucial role in maintaining the homeostasis of glomerular endothelial cells. From semi-quantitative analysis of VEGF, no difference of VEGF expression on podocytes was detected between MN cases with and without FSGS lesion (Table 2).

**Table 2:** Several histological parameters in MN cases with or without FSGS lesion.

	MN with	MN without	P
	FSGS lesion	FSGS lesion	
VEGF expression (index)**	1.2 ± 0.7	1.4 ± 0.9	0.6
global sclerotic glomeruli (%)*	11.4 ± 10.8	7.2 ± 7.1	0.1
interstitial fibrosis (index)**	1.2 ± 0.5	1.0 ± 0.6	0.06
arteriosclerosis (index)**	1.7 ± 0.8	1.8 ± 0.7	0.82

* Student’s t-test

** Mann-Whitney U test

Staining intensity of VEGF based on immunohistochemical study was scored semiquantitatively (see Materials and Methods).

Interstitial fibrosis and arteriosclerosis were also graded semiquantitatively (see Materials and Methods).

**Fig 8:**
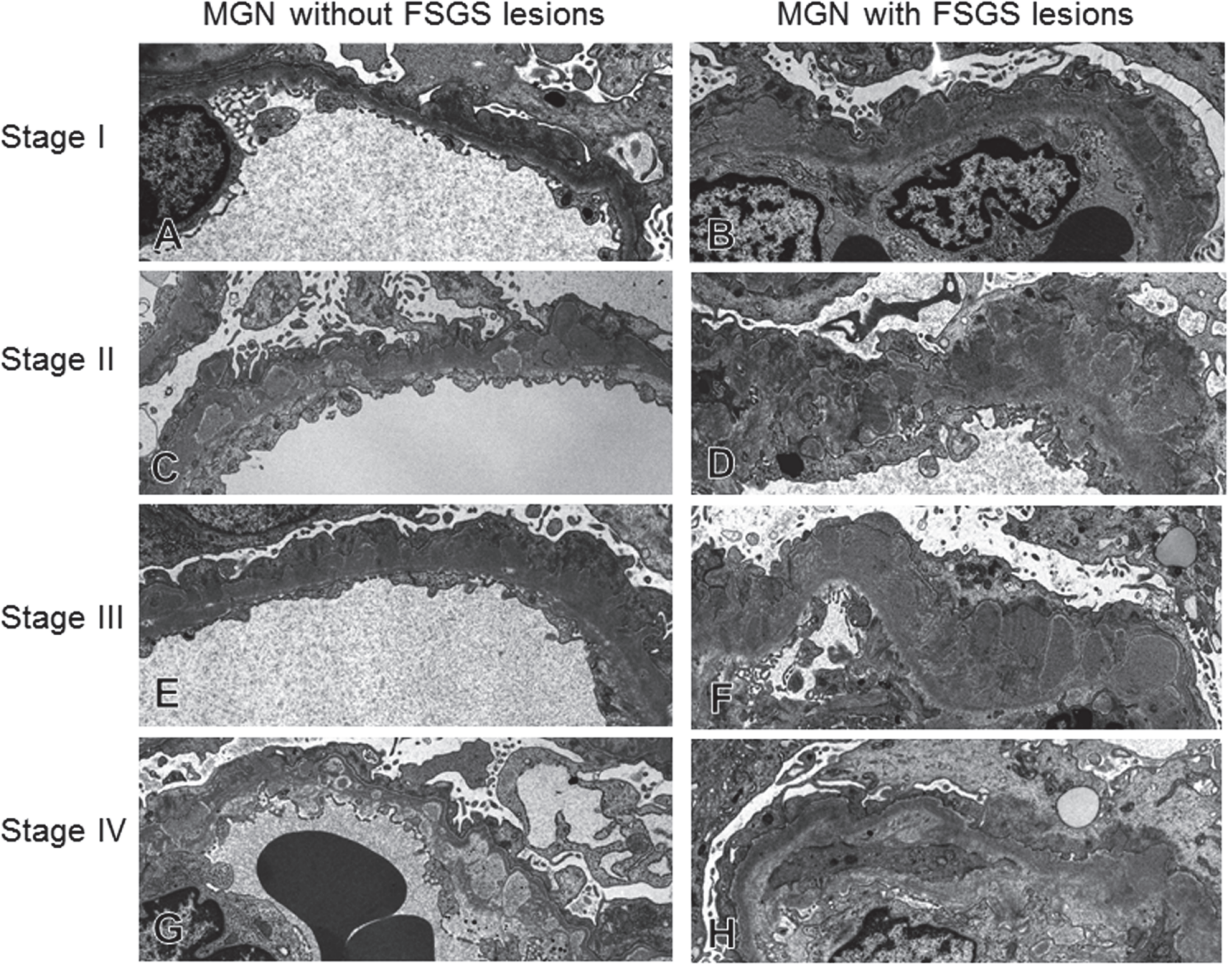
The alteration of glomerular capillaries in cases of idiopathic membranous nephropathy (MN) with/without FSGS lesion. Representative alterations of glomerular capillary walls were indicated from stage I to Stage IV of MN. The thickening of glomerular capillary walls was seen with ECM accumulation in both MN cases with and without FSGS lesion, with subepithelial deposits in stage I, spike formation in stage II to III, and wash out of deposits in stage IV. However, in MN-FSGS(−) cases, glomerular capillary walls with subepithelial deposits in stage I to IV were characterized by well-preserved fenestra of glomerular endothelial cells, less widening of subendothelial space, mild thickening of glomerular capillary walls and GBM, and relatively preserved foot processes of podocytes. On the other hand, in MN-FSGS(+) cases there were prominent thickening of glomerular capillary walls with ECM accumulation, loss of foot processes of podocytes, and endothelial cell injury, indicated by loss of fenestra, swelling of cytoplasm and dilatation of subendothelial space.

Next, we examined with electron microscope the glomerular capillary walls in more details, and measured the thickness of capillary walls in MN cases with or without FSGS lesion (Fig. 8, Table 3). The thickness of capillary wall increased more significantly in MN-FSGS(+) than in MN-FSGS(−), particularly in stages 2 to 4. In addition, the average thickness of capillary walls of all stages (total in Table 3) increased more significantly in MN-FSGS(+) than in MN-FSGS(−) cases.

**Table 3:** The thickness of glomerular capillary walls (μm) in MN cases with and without FSGS lesion.

Stage of MN		MN with FSGS lesion (μm)	MN without FSGS lesion (μm)	P
I	(n = 4)	0.75 ± 0.34	0.61 ± 0.30	0.2
II	(n = 10)	1.33 ± 0.54	0.84 ± 0.30	0.001
III	(n = 7)	1.68 ± 0.69	0.98 ± 0.39	< 0.001
IV	(n = 5)	1.80 ± 0.70	1.39 ± 0.58	0.05
Total	(n = 26)	1.48 ± 0.70	1.02 ± 0.48	< 0.001

The thickness of glomerular capillary walls was measured in electron microscopic photographs.

The thickness of glomerular capillary walls was defined as capillary walls between the bottom of podocytes and the bottom of endothelial cells, including thesubepithelial deposits, glomerular basement membrane, and subendothelial space with extracellular matrix accumulation.

Concerning the other morphological findings (Table 2), there were no significant differences in the percentage of global sclerotic glomeruli, the degree of interstitial fibrosis, and severity of arteriosclerosis between MN cases with and without FSGS lesion.

## Discussion

Glomerular endothelial cell injury is ultrastructually characterized by the narrowing of glomerular capillaries with increased number of endothelial cells in capillary lumens, swelling of cytoplasm and loss of fenestra of endothelial cells, and widening of subendothelial space and double contour of GBM with the accumulation of ECM and/or mesangial interposition. In the present study, all of these findings occurred in the glomeruli in MN cases with or without FSGS. From our results, glomerular endothelial cell injury develops in all cases of MN, regardless of the presence or absence of FSGS lesion in glomeruli.

In the present study, FSGS lesion was observed in 10.4% of MN cases, the frequency of that was slightly lower compared with the results of other studies which ranged from 12.8% to 43%[8,10–12,21]. Similar to other reports, NOS (70%) and PH (30%) lesions are the most common types of FSGS, and cellular, TIP, and collapsing lesions are very rare [8,10,21]. FSGS lesion was morphologically characterized by loss of glomerular capillaries with ECM accumulation in glomeruli, demonstrating that glomerular capillary injury may be associated with the formation of FSGS lesion. Furthermore, in computer-assisted morphometric analysis, the glomeruli in MN-FSGS(+) cases, the glomeruli had significantly smaller glomerular capillaries and larger ECM area in glomeruli than in MN-FSGS(−) cases, indicating that glomerular endothelial cell injury is more severe in MN-FSGS(+) cases. Clinically, eGFR was significantly lower in MN-FSGS(+) than in MN-FSGS(−). In the present study, we therefore concluded that glomerular endothelial cell injury develop in all cases of MN, and severe glomerular endothelial cell injury may be associated with the deterioration of eGFR and the formation of FSGS lesion in MN.

There was no difference in VEGF expression on podocytes between MN cases with and without FSGS lesion, although morphological podocyte injury developed in MN cases. However, significant difference of the thickness of glomerular capillary walls with ECM accumulation was evident between MN cases with and without FSGS lesion. We therefore concluded that glomerular endothelial injury may be associated with podocyte injury and the failure of podocyte-glomerular endothelial interaction by the thickening of capillary walls with subepithelial deposits and accumulation and organization of ECM.

The clinical course and prognosis of MN is quite variable. A number of literature discussed clinical and histologic predictive factors in MN. Clinical parameters that are poor prognostic indicators include male gender, older age, deterioration of eGFR, high levels of serum creatinine, heavy proteinuria and nephrotic syndrome, and hypertension [8,10,12,14,22]. Histologically, high incidence of global sclerotic glomeruli, presence of glomerular FSGS lesion, high degree of interstitial fibrosis, and severe arteriosclerosis have been considered poor prognostic factors in MN [8,10,11,14,23]. In the present study, although we did not examine the prognosis of MN, eGFR at the time of biopsy was significant lower in MN-FSGS(+) than MN-FSGS(−). Meanwhile, there were no significant differences between MN with and without FSGS in other clinical risk factors such as gender, age, proteinuria, and blood pressure. In addition, there were no significant differences in histological parameters, such as the incidence of global sclerotic glomeruli, the degree of interstitial fibrosis, and severity of arteriosclerosis except for the presence of glomerular FSGS. Our results indicate that the coexistence of FSGS lesion in MN is most likely associated with renal insufficiency without other clinical and histologic risk factors.

This study analyzed glomerular endothelial cell injury in MN-FSGS(+) cases, which has not been done in the past, and demonstrated that glomerular capillary injury was due to endothelial cell damage and ECM accumulation in MN. In addition, the prominent injury of glomerular capillaries may be associated with the formation of FSGS lesion in MN. We considered three possible mechanisms of glomerular capillary injury in MN: 1) the development of glomerular hypertrophy, 2) podocyte injury and the decreased expression of VEGF of podocytes, and 3) the thickening of glomerular capillary walls with ECM accumulation.

In regards to glomerular hypertrophy in MN, Hughson et al. [24] reported that glomeruli of MN were significantly larger in size than in other diseases such as primary FSGS, minimal change disease, lupus nephritis, mesangial proliferative glomerulonephritis, hypertensive nephropathy, and diabetic nephropathy. In the present study with our computer-assisted morphometric analysis, the glomeruli of MN with or without FSGS lesion were larger in size compared to glomeruli in control cases with minor glomerular abnormalities. However, no significantly difference of the glomerular hypertrophy was evident between MN cases with and without FSGS lesion. We therefore concluded that glomerular hypertrophy was not associated with the formation of FSGS lesion in MN.

Next, we focused on podocyte injury and the expression of VEGF on glomerular podocytes. Several recent studies suggested that subepithelial immune deposits in MN may lead to disruption of the podocyte attachment to the GBM, a phenomenon observed in primary FSGS, and contribute to develop the FSGS [8,11,21]. Indeed, podocytes in urine are slightly increased in some MN cases [25]. In glomerular diseases, apical cell membranes of injured podocytes can be shed into the urine as podocalyxin-positive granular structures in urinary sediments, and urinary podocalyxin is a useful biomarker of podocyte injury [26,27]. In addition, recent clinical studies demonstrated that the expression of VEGF in glomerular podocytes is diminished in active MN with decreased expression of urinary VEGF [28,29]. VEGF, produced mainly by glomerular podocytes, has a crucial role in maintaining the homeostasis of glomerular endothelial cells and a protective and reparative role in injury of glomerular endothelial cells, promoting survival, proliferation, and differentiation of endothelial cells [30]. In glomerular diseases, VEGF-expressing cells were decreased in number or absent in areas of focal or global glomerular sclerosis [31]. Avihingsanon et al. demonstrated in an experimental model that VEGF is required for glomerular and tubular hypertrophy and proliferation in response to nephron reduction, and down-regulation of VEGF is associated with the development of glomerulosclerosis and tubulointerstitial fibrosis in the remnant kidney [32]. In our ultrastructual study, podocyte injury developed with effacement of foot processes in MN. In addition, podocyte injury in MN-FSGS(+) cases was more prominent than those in MN-FSGS(−) cases. In the present study, although there are limitations to semi-quantitative analysis of VEGF in tissue sections, no difference of VEGF expression on podocytes was detected between MN cases with and without FSGS lesion.

We finally focused on thickness of glomerular capillary walls that may influence the function of VEGF acting on glomerular endothelial cells. Yoshimoto et al. had indicated that an electron microscopic classification of heterogeneous type or deep subgroup type with electron dense deposit with thickening capillary walls are independent risk factors in MN [23]. Lee et al. observed that increased thickening of the GBM is more frequently present in MN cases with FSGS lesion [11]. They also indicated that the advanced thickening of GBM and occurrence of FSGS are associated with advanced stage of MN. In the present study, the thickness of glomerular capillary walls increased more significantly in MN-FSGS(+) than in MN-FSGS(−) cases. Recent reports suggest that VEGF produced by the podocyte is transported to glomerular endothelilal cells across the GBM by diffusion [33,34], and this paracrine signaling of VEGF is critical for maintaining the function of glomerular endothelial cells and the glomerular filtration barrier [35]. We therefore concluded that the significant thickening of glomerular capillary walls with subepithelial deposits and ECM accumulation may influence the function of VEGF from podocytes, resulting in the glomerular capillary endothelial cell injury that contribute to the development of FSGS lesion in MN.

Further investigations are necessary concerning the relationship between glomerular endothelial cell injury and the formation of FSGS, lesion development of renal insufficiency, and the mechanism of glomerular endothelial injury in MN.

## References

[1] Haas M, Meehan SM, Karrison TG, Spargo BH. Changing etiologies of unexplained adult nephrotic syndrome: a comparison of renal biopsy findings from 1976–1979 and 1995–1997. Am J Kidney Dis. 1997;30: 621–631. 9370176 10.1016/s0272-6386(97)90485-6

[2] Fervenza FC, Sethi S, Specks U. Idiopathic membranous nephropathy: diagnosis and treatment. Clin J Am Soc Nephrol. 2008;3: 905–919. 10.2215/CJN.04321007 18235148

[3] Cattran DC. Idiopathic membranous glomerulonephritis. Kidney Int. 2001;59: 1983–1994. 11318974 10.1046/j.1523-1755.2001.0590051983.x

[4] Cattran DC, Pei Y, Greenwood C. Predicting progression in membranous glomerulonephritis. Nephrol Dial Transplant. 1992;7 Suppl 1: 48–52. 1337182

[5] Cattran DC, Pei Y, Greenwood CM, Ponticelli C, Passerini P, Honkanen E. Validation of a predictive model of idiopathic membranous nephropathy: its clinical and research implications. Kidney Int. 1997;51: 901–907. 9067928 10.1038/ki.1997.127

[6] Reichert LJ, Koene RA, Wetzels JF. Prognostic factors in idiopathic membranous nephropathy. Am J Kidney Dis. 1998;31: 1–11. 9428445 10.1053/ajkd.1998.v31.pm9428445

[7] Schieppati A, Mosconi L, Perna A, Mecca G, Bertani T, Garattini S. Prognosis of untreated patients with idiopathic membranous nephropathy. N Engl J Med. 1993;329: 85–89. 8510707 10.1056/NEJM199307083290203

[8] Dumoulin A, Hill GS, Montseny JJ, Meyrier A. Clinical and morphological prognostic factors in membranous nephropathy. Am J Kidney Dis.2003;41: 38–48. 12500220 10.1053/ajkd.2003.50015

[9] Van Damme B, Tardanico R, Vanrenterghem Y, Desmet V. Adhesions, focal sclerosis, protein crescents, and capsular lesions in membranous nephropathy. J Pathol. 1990;161: 47–56. 2370598 10.1002/path.1711610109

[10] Wakai S, Magil AB. Focal glomerulosclerosis in idiopathic membranous glomerulonephritis. Kidney Int. 1992;41: 428–434. 1552716 10.1038/ki.1992.59

[11] Lee HS, Koh HI. Nature of progressive glomerulosclerosis in human membranous nephropathy. Clin Nephrol. 1993;39: 7–16. 8428410

[12] Heeringa SF, Branten AJ, Deegens JK, Steenbergen E, Wetzels JF. Focal segmental glomerulosclerosis is not a sufficient predictor of renal outcome in patients with membranous nephropathy. Nephrol Dial Transplant. 2007;22: 2201–2207. 17442739 10.1093/ndt/gfm188

[13] Troyanov S, Roasio L, Pandes M, Herzenberg AM, Cattran DC. Renal pathology in idiopathic membranous nephropathy: a new perspective. Kidney Int. 2006;69: 1641–1648. 16572119 10.1038/sj.ki.5000289

[14] Shiiki H, Saito T, Nishitani Y, Mitarai T, Yorioka N, et al Prognosis and risk factors for idiopathic membranous nephropathy with nephrotic syndrome in Japan. Kidney Int. 2004;65: 1400–1407. 15086481 10.1111/j.1523-1755.2004.00518.x

[15] Barisoni L, Schnaper HW, Kopp JB. A proposed taxonomy for the podocytopathies: a reassessment of the primary nephrotic diseases. Clinical Journal of the American Society of Nephrology. 2007;2: 529–542. 17699461 10.2215/CJN.04121206

[16] Machado JR, Rocha LP, Neves PD, Cobô EeC, Silva MV, Castellano LR, et al An overview of molecular mechanism of nephrotic syndrome. Int J Nephrol. 2012;2012: 937623 10.1155/2012/937623 22844593 PMC3401527

[17] Nochy D, Heudes D, Glotz D, Lemoine R, Gentric D, Bruneval P, et al Preeclampsia associated focal and segmental glomerulosclerosis and glomerular hypertrophy: a morphometric analysis. Clin Nephrol. 1994;42: 9–17. 7923975

[18] Nishimoto K, Shiiki H, Nishino T, Kimura T, Sasaki Y, Yamasaki M, et al Glomerular hypertrophy in preeclamptic patients with focal segmental glomerulosclerosis. A morphometric analysis. Clin Nephrol. 1999;51: 209–219. 10230553

[19] D'Agati VD, Fogo AB, Bruijn JA, Jennette JC. Pathologic classification of focal segmental glomerulosclerosis: a working proposal. Am J Kidney Dis. 2004;43: 368–382. 14750104 10.1053/j.ajkd.2003.10.024

[20] Ehrenreich T, Churg J. Pathology of membranous nephropathy. Pathol Annu. 1968;3: 145–186.

[21] Gupta R, Sharma A, Mahanta PJ, Jacob TG, Agarwal SK, Roy TS, et al Focal segmental glomerulosclerosis in idiopathic membranous glomerulonephritis: a clinico-pathological and stereological study. Nephrol Dial Transplant. 2010;25: 444–449. 10.1093/ndt/gfp521 19808947

[22] Polanco N, Gutiérrez E, Covarsí A, Ariza F, Carreño A, Vigil A, et al Spontaneous remission of nephrotic syndrome in idiopathic membranous nephropathy. J Am Soc Nephrol. 2010;21: 697–704. 10.1681/ASN.2009080861 20110379 PMC2844306

[23] Yoshimoto K, Yokoyama H, Wada T, Furuichi K, Sakai N, Iwata Y, et al Pathologic findings of initial biopsies reflect the outcomes of membranous nephropathy. Kidney Int. 2004;65: 148–153. 14675045 10.1111/j.1523-1755.2004.00403.x

[24] Hughson MD, Johnson K, Young RJ, Hoy WE, Bertram JF. Glomerular size and glomerulosclerosis: relationships to disease categories. Am J Kidney Dis. 2002 39: 679–688. 11920332 10.1053/ajkd.2002.31980

[25] Hara M, Yanagihara T, Kihara I. Urinary podocytes in primary focal segmental glomerulosclerosis. Nephron. 2001;89: 342–347. 11598401 10.1159/000046097

[26] Hara M, Yanagihara T, Kihara I, Higashi K, Fujimoto K, Kajita T. Apical cell membranes are shed into urine from injured podocytes: a novel phenomenon of podocyte injury. J Am Soc Nephrol. 2005;16: 408–416. 15625073 10.1681/ASN.2004070564

[27] Hara M, Yanagihara T, Hirayama Y, Ogasawara S, Kurosawa H, Sekine S, et al Podocyte membrane vesicles in urine originate from tip vesiculation of podocyte microvilli. Hum Pathol. 2010;41: 1265–1275. 10.1016/j.humpath.2010.02.004 20447677

[28] Honkanen EO, Teppo AM, Grönhagen-Riska C. Decreased urinary excretion of vascular endothelial growth factor in idiopathic membranous glomerulonephritis. Kidney Int. 2000;57: 2343–2349. 10844604 10.1046/j.1523-1755.2000.00094.x

[29] Honkanen E, von Willebrand E, Koskinen P, Teppo AM, Törnroth T, Ruutu M, et al Decreased expression of vascular endothelial growth factor in idiopathic membranous glomerulonephritis: relationships to clinical course. Am J Kidney Dis. 2003;42: 1139–1148. 14655184 10.1053/j.ajkd.2003.08.014

[30] Schrijvers BF, Flyvbjerg A, De Vriese AS. The role of vascular endothelial growth factor (VEGF) in renal pathophysiology. Kidney Int. 2004;65: 2003–2017. 15149314 10.1111/j.1523-1755.2004.00621.x

[31] Shulman K, Rosen S, Tognazzi K, Manseau EJ, Brown LF. Expression of vascular permeability factor (VPF/VEGF) is altered in many glomerular diseases. J Am Soc Nephrol. 1996;7: 661–666. 8738799 10.1681/ASN.V75661

[32] Avihingsanon Y, Benjachat T, Tassanarong A, Sodsai P, Kittikovit V, Hirankarn N. Decreased renal expression of vascular endothelial growth factor in lupus nephritis is associated with worse prognosis. Kidney Int. 2009;75: 1340–1348. 10.1038/ki.2009.75 19295501

[33] Eremina V, Jefferson JA, Kowalewska J, Hochster H, Haas M, Weisstuch J. VEGF inhibition and renal thrombotic microangiopathy. N Engl J Med. 2008;358: 1129–1136. 10.1056/NEJMoa0707330 18337603 PMC3030578

[34] Katavetin P. VEGF inhibition and renal thrombotic microangiopathy. N Engl J Med. 2008;359: 205–206; author reply 206–207. 10.1056/NEJMc080770 18614790

[35] Sison K, Eremina V, Baelde H, Min W, Hirashima M, Fantus IG. Glomerular structure and function require paracrine, not autocrine, VEGF-VEGFR-2 signaling. J Am Soc Nephrol. 2010;21: 1691–1701. 10.1681/ASN.2010030295 20688931 PMC3013545

